# Reoperation, Readmission, and Postoperative Bleeding in Pediatric Cerebral Palsy Patients Undergoing Spinal Arthrodesis

**DOI:** 10.7759/cureus.62520

**Published:** 2024-06-17

**Authors:** Michael J Miskiewicz, Shabnam Parsa, Matthew Magruder, Amr Abdelgawad

**Affiliations:** 1 Department of Orthopaedic Surgery, Stony Brook University, Stony Brook, USA; 2 Department of Surgery, Stony Brook University, Stony Brook, USA; 3 Department of Orthopedic Surgery, Maimonides Medical Center, Brooklyn, USA

**Keywords:** orthopedic surgery, pediatric surgery, postoperative outcomes, spine surgery, cerebral palsy (cp)

## Abstract

Background

Cerebral palsy (CP) is one of the most common neuromuscular disorders in children, and spinal abnormalities are vastly more common in people with CP compared to the general population. Further investigation is needed to improve our understanding of the perioperative factors that place children with CP at greater risk of postoperative complications. This study aims to investigate (1) whether pediatric CP patients have higher rates of postoperative complications after spinal fusion and (2) risk factors for postoperative bleeding, readmission, and reoperation.

Methodology

The 2019 American College of Surgeons National Surgical Quality Improvement Program Pediatric database was used for this study. Chi-square tests were used to compare patient demographics, frequency of comorbidities, intraoperative factors, and postoperative complications between CP and non-CP patients. Multivariable logistic regression modeling was conducted to determine if CP was an independent risk factor for the composite variable that included postoperative bleeding, readmission, and reoperation.

Results

A total of 4,445 patients were included in the study, with 606 CP and 3,839 non-CP patients. Several comorbidities were more prevalent in the CP cohort, most notably asthma, gastrointestinal disease, previous cardiac surgery, and hematologic disorders. Multivariable logistic regression modeling revealed that CP, older age, non-Caucasian race, American Society of Anesthesiologists (ASA) class of 3 or higher, posterior surgical approach, previous cardiac surgery, and ostomy were significantly correlated with higher postoperative complications.

Conclusions

This study demonstrates that CP, older age, non-Caucasian race, ASA class of 3 or higher, posterior approach, previous cardiac surgery, and ostomy are independent risk factors for postoperative complications, including readmission, reoperation, and postoperative bleeding requiring transfusions. Consequently, there is a pressing need for additional research to establish perioperative strategies that reduce postoperative risks for these patients. Spine surgeons should consider the findings of this study when communicating the potential risks of spinal fusion surgery with patients and their families.

## Introduction

Cerebral palsy (CP) is a non-progressive neurodevelopmental disorder affecting various aspects of motor function [1-3]. With a prevalence of 2-2.5 cases per 1,000 births, CP is the most common childhood physical disability [1,4-6]. Because of its associated heterogeneity, CP motor impairments are variable due to inconstant injury of the developing fetal or infant brain [6,7]. CP patients are also often vulnerable to a variety of comorbidities. The quality of life of CP patients is directly associated with appropriate interdisciplinary management of these comorbidities as they progress throughout development [8]. As a result, surgery is often recommended for patients with CP to treat these coexisting disorders.

Scoliosis remains one of the most prevalent and consequential comorbidities associated with CP syndrome [3,9,10]. Pelvic obliquity and progressive trunk imbalance caused by scoliosis induce ischial weight-bearing stress in seated positions for CP patients [9]. Moreover, degrees of scoliotic curvatures progressively worsen with age [10], often to the point where spinal fusion is required. This intervention aims to provide patients with a well-positioned pelvis and a well-balanced trunk [10]. Nevertheless, the morbidity burden of CP patients with scoliosis results in an increased risk of spinal fusion postoperative complications [11]. These complications are broadly classified into infectious and non-infectious outcomes.

Assessment of non-infectious complications of spinal arthrodesis could highlight areas of discrepancy for pediatric CP patients. There is a limited analysis of postoperative complications for pediatric CP patients [12]. Therefore, we aim to identify and evaluate the preoperative and intraoperative risk factors that predispose pediatric CP patients to increased rates of readmission, reoperation, and postoperative bleeding requiring transfusions.

## Materials and methods

The American College of Surgeons National Surgical Quality Improvement Program (ACS NSQIP) Pediatric maintains a nationally recognized and adjusted database that quantifies postoperative outcomes within 30 days for various surgical procedures. The database, which has been widely used in research on surgical outcomes for adult and pediatric patients, is prospectively updated from nearly 60 hospitals and validated by the ACS NSQIP. Clinical information is collected by trained professionals, and a rigorous auditing process ensures the accuracy of the data. This study utilized the 2019 annual database from the ACS NSQIP Pediatric. As the database does not publish any information that could identify patients, providers, or hospitals, it was exempt from our institutional review board approval.

For this study, the records of pediatric patients who underwent spinal fusion surgery in 2019 (January 1st to December 31st) were analyzed through the ACS NSQIP Pediatric database. The procedure of anterior arthrodesis was indicated by the CPT codes 22808, 22810, and 22812, while the codes 22800, 22802, and 22804 signified posterior arthrodesis. All previous codes were included in our search. Patients were then divided into the following two groups: the CP group and the non-CP group. Individuals with incomplete or missing data were excluded from the analysis.

The initial phase of the study involved gathering information on patient demographics, comorbidities, and various intraoperative factors. If significant differences were found between the cohorts, these perioperative predictors were included in the multivariable analysis. To serve as the dependent variable in the multivariable logistic regression, a composite variable was created to indicate whether patients experienced at least one of the following complications: postoperative bleeding requiring transfusion, readmission, or reoperation.

The study compared the differences in patient demographics, preoperative comorbidities, intraoperative factors, and frequency of postoperative morbidity and mortality between the CP and non-CP groups using chi-square tests. Variables with a p-value of less than 0.05 were considered statistically significant and were included in the multivariate analysis. To determine which perioperative factors were independent risk factors for the previously described composite postoperative variable, likelihood ratio tests were used in multivariable logistic regression modeling.

## Results

A preliminary review of the NSQIP Pediatric database revealed a total of 4,445 patients who underwent spinal fusion surgery in 2019. Of these, 606 (13.63%) had CP, and 3,839 (86.37%) did not. The CP cohort had significantly different demographics, including a lower mean age (CP = 12.98 vs. non-CP = 13.89) and a higher percentage of males (49.01% CP vs. 26.75% non-CP), Caucasians (59.41% CP vs. 65.59% non-CP Caucasian), and Hispanics (17.33% CP vs. 11.70% non-CP) (Table 1).

**Table 1:** Baseline demographic profiles of pediatric patients with and without CP undergoing spinal fusion surgery. CP = cerebral palsy

Demographic characteristics	CP		No CP		
	n	Percentages	n	Percentages	P-value
Total (4,445)	606	13.63	3,839	86.37	-
Age, mean/SD	12.98	2.79	13.89	2.73	<0.001
Gender					
Male	297	49.01	1027	26.75	<0.001
Female	309	50.99	2812	73.25	
Race					
American Indian or Alaska Native	5	0.83	8	0.21	<0.001
Asian	14	2.31	120	3.13	
Black or African American	122	20.13	577	15.03	
Native Hawaiian or Other Pacific Islander	3	0.50	4	0.10	
Unknown/Not reported	102	16.83	612	15.94	
White	360	59.41	2518	65.59	
Ethnicity					
Hispanic	105	17.33	449	11.70	<0.001
Non-Hispanic	441	72.77	2979	77.60	
N/A	60	9.90	411	10.71	

Univariate analysis found that CP patients were significantly more likely to have coexisting medical conditions at the time of surgery, such as ventilator dependence (8.58% vs. 1.09%, p < 0.001), asthma (22.11% vs. 5.97%, p < 0.001), bronchopulmonary dysplasia (22.61% vs. 2.03%, p < 0.001), oxygen support (9.08% vs. 0.70%, p < 0.001), tracheostomy (10.73% vs. 0.68% p < 0.001), structural pulmonary abnormality (20.79% vs. 4.01%, p < 0.001), gastrointestinal disease (44.22% vs. 4.58%, p < 0.001), previous cardiac surgery (5.61% vs. 3.02% p = 0.001), nutritional support (50.99% vs. 1.93%, p < 0.001), hematologic disorder (6.44% vs. 2.16%, p < 0.001), steroid use (2.15% vs. 0.68%, p < 0.001), ostomy (61.22% vs. 2.89%, p < 0.001), intraventricular hemorrhage (7.10% vs. 0.23% p < 0.001), and cardiac risk factors (12.87% vs. 7.29%, p < 0.001) (Table 2). Additionally, CP patients underwent posterior arthrodesis (96.86% vs. 94.95%, p = 0.04), were discharged to a location other than their home (9.57% vs. 0.96%, p < 0.001), and required surgery that was classified in the database as urgent or emergent, as opposed to elective surgery (1.98% vs. 0.73%, p = 0.002) more frequently than patients without CP (Table 3). CP patients experienced greater rates of bleeding requiring transfusions (85.64% vs. 64.94%, p < 0.001), readmissions (10.73 vs. 2.68%, p < 0.001), and reoperations (6.60% vs. 1.85%, p < 0.001) (Table 4).

**Table 2:** Preoperative comorbidities and ASA classification of patients with and without CP undergoing spinal fusion surgery. ASA = American Society of Anesthesiologists; CP = cerebral palsy

Preoperative factors	CP		No CP		
	n	Percentages	n	Percentages	P-value
ASA class					
ASA 1	4	0.66	733	19.09	<0.001
ASA 2	45	7.43	2305	60.04	
ASA 3	508	83.83	753	19.61	
ASA 4	46	7.59	26	0.68	
ASA not assigned	3	0.50	22	0.57	
Comorbidities					
Ventilator dependence	52	8.58	42	1.09	<0.001
Asthma	134	22.11	229	5.97	<0.001
Bronchopulmonary dysplasia	137	22.61	78	2.03	<0.001
Oxygen support	55	9.08	27	0.70	<0.001
Tracheostomy	65	10.73	26	0.68	<0.001
Structural pulmonary abnormalities	126	20.79	154	4.01	<0.001
Gastrointestinal disease	268	44.22	176	4.58	<0.001
Inotropic support at the time of surgery	2	0.33	20	0.52	0.534
Previous cardiac surgery	34	5.61	116	3.02	0.001
Nutritional support	309	50.99	74	1.93	<0.001
Hematologic disorder	39	6.44	83	2.16	<0.001
Steroid use	13	2.15	26	0.68	<0.001
Ostomy	371	61.22	111	2.89	<0.001
Intraventricular hemorrhage	43	7.10	9	0.23	<0.001
Cardiac risk factors	78	12.87	280	7.29	<0.001
Sepsis/Septic shock 48 hours prior to surgery	1	0.17	15	0.39	0.389

**Table 3:** Characteristics of the surgery performed and the location where the patient was discharged.

Operative factors	Cerebral palsy		No cerebral palsy		
	n	Percentages	n	Percentages	P-value
Location of surgery					
Inpatient	587	96.86	3745	97.55	0.318
Outpatient	19	3.14	94	2.45	
Surgical approach					
Anterior arthrodesis	19	3.14	194	5.05	0.040
Posterior arthrodesis	587	96.86	3645	94.95	
Discharge location					
Home	548	90.43	3802	99.04	<0.001
Not home	58	9.57	37	0.96	
Surgical technique					
Laparoscopic ± open	6	0.99	74	1.93	0.107
Open only	600	99.01	3765	98.07	
Case type					
Elective	594	98.02	3811	99.27	0.002
Emergent/Urgent	12	1.98	28	0.73	

**Table 4:** Postoperative complications and composite variable.

Postoperative morbidities	Cerebral palsy		No cerebral palsy		
	n	Percentages	n	Percentages	P-value
Composite variable	416	68.65	2637	68.69	<0.001
Bleeding/Transfusion	519	85.64	2493	64.94	<0.001
Readmission	65	10.73	103	2.68	<0.001
Reoperation	40	6.60	71	1.85	0.005

Multivariable logistic regression modeling revealed that CP (odds ratio (OR) = 2.62, confidence interval (CI) = 1.93-3.55, p < 0.001), older age (OR = 1.11, CI = 1.08-1.14, p < 0.001), non-Caucasian race as opposed to Caucasian race (OR = 0.74, CI = 0.64-0.86, p < 0.001), American Society of Anesthesiologists (ASA) class of 3 or higher (OR = 1.26, CI = 1.03-1.54, p = 0.022), posterior as opposed to anterior surgical approach (OR = 0.21, CI = 0.15-0.29, p < 0.001), history of previous cardiac surgery (OR = 1.84, CI = 1.12-3.05, p = 0.017), and ostomy (OR = 1.80, CI = 1.16-2.79, p = 0.009) were significantly correlated with higher postoperative complications (Table 5).

**Table 5:** Multivariable logistic regression comparing risk factors for any infectious complication composite variable. OR = odds ratio; CI = 95% confidence interval

Factor	OR	CI	P-value
Cerebral palsy	2.62	1.93–3.55	<0.001
Age	1.11	1.08–1.14	<0.001
Body mass index	1.00	0.99–1.01	0.581
Sex	1.11	0.95–1.30	0.182
Race (White vs. non-White)	0.74	0.64–0.86	<0.001
American Society of Anesthesiologists class >3	1.26	1.03–1.54	0.022
Surgical approach (anterior vs. posterior)	0.21	0.15–0.29	<0.001
Case type (elective vs. urgent/emergent)	0.56	0.27–1.18	0.126
Bronchopulmonary dysplasia	1.20	0.79–1.84	0.396
Structural pulmonary abnormality	0.99	0.70–1.39	0.931
Gastrointestinal disease	0.91	0.67–1.23	0.525
Cardiac risk factors	0.76	0.55–1.05	0.101
Asthma	1.16	0.87–1.54	0.308
Previous cardiac surgery	1.84	1.12–3.05	0.017
Hematologic disorder	1.51	0.93–2.47	0.097
Ventilator	0.99	0.57–1.74	0.976
Oxygen support	1.37	0.74–2.52	0.319
Tracheostomy	1.00	0.56–1.78	0.993
Steroid	1.01	0.47–2.17	0.983
Ostomy	1.80	1.16–2.79	0.009
Nutritional support	0.66	0.42–1.05	0.081
Days from hospital admission to operation	0.99	0.98–1.00	0.054
Duration: anesthesia start to surgery start	0.99	0.99–1.00	0.015
Duration: surgery stop to anesthesia stop	1.00	1.00–1.01	0.520
Time the patient is in the operating room	1.00	0.99–1.00	0.131
Total anesthesia time	1.00	1.00–1.01	0.423
Total operation time	1.01	1.00–1.01	0.001

## Discussion

This study used the ACS NSQIP Pediatric database to study the relative risk profiles of pediatric patients with and without CP. In addition, specific risk factors in pediatric CP patients that result in increased postoperative bleeding, readmission, and reoperation following spinal fusion surgery were evaluated. We found that compared to children without CP, the following risk factors were correlated with increased complication rates following spinal fusion surgery in the CP patient population: older age, non-Caucasian race vs. Caucasian race, ASA class of 3 or higher, posterior approach vs. anterior approach, previous cardiac surgery, and ostomy.

The prevalence of neuromuscular scoliosis in patients with CP far exceeds that of the general population [1,4,5], and proper therapeutic intervention is key to preventing the progression of scoliotic curvatures and additional functional impairment in these patients. Management of CP patients with scoliosis utilizes several operative and non-operative options; however, spinal fusion surgery is often reported by patients and caregivers as the most beneficial intervention for improving functional outcomes and overall quality of life [13]. While surgical intervention is justified and recommended to many of these patients, the risk of postoperative complications should be adequately explained by surgeons to CP patients and caregivers. Complications of neurological, respiratory, and gastrointestinal function are among the more common manifestations of postoperative morbidity and mortality in CP patients after surgery [14-16]. The breadth of research investigating the relationship between CP and postoperative complications in a solely pediatric population is limited. Here, we identify CP and other key independent risk factors of increased risk for readmission, reoperation, and postoperative bleeding requiring transfusion(s) after spinal fusion surgery in a pediatric population.

Our results suggest that a CP diagnosis before spinal fusion surgery in pediatric patients is an independent risk factor for increased postoperative bleeding, reoperation, and readmission. Previous research that identified worse postoperative outcomes in CP patients has often explained this observation by simply noting that CP patients often present with more comorbidities and poorer overall health compared to the general population [8,12,17]. Here, we posit that a more comprehensive explanation is warranted to better understand this variance in post-spinal fusion outcomes. For example, we found that CP patients were discharged to a location other than their home more frequently than the non-CP cohort (9.57% vs. 0.96%). Discharge to a skilled or unskilled nursing facility could place CP patients at a greater risk of inadequate care or surgical-site community-acquired infections. CP patients with limited motor and language abilities may have difficulty communicating with assisted living facility staff as well. This potentially creates a situation where postoperative recommendations by the surgeon may not be properly followed.

A history of previous cardiac surgery, ostomy, and ASA class 3 or greater are also found to be significant independent risk factors for postoperative bleeding, readmission, and reoperation following spinal fusion surgery. These findings highlight the importance of using a comprehensive approach to preoperative risk assessment that takes into account a patient’s overall health status and surgical history. For instance, a history of cardiac surgery may imply the recent use of blood thinners and an overall state of weakened cardiovascular health, which can increase the risk of bleeding during spinal fusion surgery. Similarly, ostomy tube placement and gastrointestinal health can also play a role in the risk of postoperative complications, as they may be indicative of underlying gastrointestinal disorders or poor nutritional status. Therefore, it is crucial to carefully evaluate a patient’s overall health status and surgical history before undergoing spinal fusion surgery to identify potential risk factors and optimize patient care accordingly. This may involve implementing measures such as optimizing nutrition, adjusting medication regimens, and closely monitoring patients during and after surgery.

Prior research has suggested other factors to be significant predictors of postoperative hospital readmission or reoperation, such as wound dehiscence, surgical site infection, dehydration, feeding issues, and acute respiratory failure [18]. Our study did not show these factors to be statistically significant on univariate analysis and were therefore not included in multivariate regression analysis. Future research may look to further characterize the prevalence of these postoperative complications in the pediatric CP population, and how they modulate hospital readmission, reoperation, and postoperative bleeding necessitating transfusions.

Critical evaluation of our study’s methods suggests that several notable limitations exist. These limitations are in part due to our reliance on the ACS NSQIP Pediatric database. One limitation is that this database only records patient outcomes for up to 30 days following surgery, and therefore, we were unable to account for any complications that may have arisen after this period. Moreover, certain variables provided in the database are generic in nature, which posed challenges to our ability to draw specific conclusions regarding their impact on postoperative outcomes. Despite these limitations, we made every effort to ensure the accuracy and validity of our findings, and we believe that our study provides important insights into the risk factors associated with spinal fusion surgery in pediatric patients. Future studies may benefit from incorporating additional data sources and more comprehensive data collection methods to further expand our understanding of these risk factors and their impact on patient outcomes.

## Conclusions

Using the ACS NSQIP Pediatric national surgical database, this study demonstrated that CP is an independent risk factor for postoperative bleeding that requires at least one transfusion, readmission, and reoperation following spinal fusion surgery. Older age, non-Caucasian race vs. Caucasian race, ASA class of 3 or higher, posterior approach vs. anterior approach, previous cardiac surgery, ostomy, shorter time from anesthesia start to surgery start, and longer total operation time were also shown to be risk factors for these postoperative complications. Orthopedic surgeons may utilize these findings during preoperative planning to minimize the risks posed to pediatric patients during recovery from spinal fusion surgery.
